# Variable seed bed microsite conditions and light influence germination in Australian winter annuals

**DOI:** 10.1007/s00442-021-05091-7

**Published:** 2022-01-09

**Authors:** Isaac R. Towers, David J. Merritt, Todd E. Erickson, Margaret M. Mayfield, John M. Dwyer

**Affiliations:** 1 School of Biological Sciences, The University of Queensland, St Lucia, QLD Australia; 2 School of Biological Sciences, The University of Western Australia, Crawley, WA Australia; 3 Department of Biodiversity, Conservation and Attractions, Kings Park Science, Perth, WA Australia

**Keywords:** Photoblasticity, Spatial coexistence, York gum-jam, Dormancy, Mediterranean

## Abstract

Environmentally cued germination may play an important role in promoting coexistence in Mediterranean annual plant systems if it causes niche differentiation across heterogeneous microsite conditions. In this study, we tested how microsite conditions experienced by seeds in the field and light conditions in the laboratory influenced germination in 12 common annual plant species occurring in the understorey of the York gum-jam woodlands in southwest Western Australia. Specifically, we hypothesized that if germination promotes spatial niche differentiation, then we should observe species-specific germination responses to light. In addition, we hypothesized that species’ laboratory germination response may depend on the microsite conditions experienced by seeds while buried. We tested the laboratory germination response of seeds under diurnally fluctuating light and complete darkness, which were collected from microsites spanning local-scale environmental gradients known to influence community structure in this system. We found that seeds of 6 out of the 12 focal species exhibited significant positive germination responses to light, but that the magnitude of these responses varied greatly with the relative light requirement for germination ranging from 0.51 to 0.86 for these species. In addition, germination increased significantly across a gradient of canopy cover for two species, but we found little evidence to suggest that species’ relative light requirement for germination varied depending on seed bank microsite conditions. Our results suggest that variability in light availability may promote coexistence in this system and that the microsite conditions seeds experience in the intra-growing season period can further nuance species germination behaviour.

**Supplementary Information:**

The online version contains supplementary material available at 10.1007/s00442-021-05091-7.

## Introduction

Mediterranean-climate regions are characterized by hot, dry summers and cool, wet winters, and support disproportionally high levels of global vascular plant diversity relative to their land area (Kreft and Jetz 2007). Plant species in these regions typically exhibit an ecological strategy which permits them to either avoid or tolerate seasonal droughts (Bernhardt 2007). For example, winter annual plants, which are a significant component of the diversity in Mediterranean ecosystems, capitalize on the winter months throughout the vegetative phase of their life cycle and avoid unfavourable conditions during the dry summer months as seeds in the seed bank (Cowling et al. 1996). Environmentally cued germination is an important adaptation to the life cycle of annual plants to ensure that the transition between seed and germinant occurs when the environment is most favorable for post-germination survival and growth (Baskin and Baskin 2014a; Donohue et al. 2010).

Seed dormancy and germination traits cue seed recruitment to the right time of year (Gremer et al. 2016), but recruitment from the seed bank is also known to vary spatially, depending on local microsite conditions (Facelli et al. 2005; Rice 1985). Indeed, spatial variation in species’ seed germination is often invoked to explain how diversity is maintained in ecological communities, because it is another axis along which species can partition their niches (i.e., the “regeneration niche”; Chesson 2000b; Grubb 1977). Fundamentally, this partitioning depends on seeds of different species exhibiting unique germination behaviour in response to different microsite conditions.

Light availability is one the major environmental factors that is perceptible by seeds and is often required to elicit germination in annual plants (Carta et al. 2017; Grime et al. 1981; Scott and Morgan 2012). However, the effect of light on the probability of germination is not consistent across species or ecosystems. On one hand, many studies have shown that a greater fraction of small-seeded species, in particular, tend to germinate under light rather than dark conditions (i.e., positive photoblasticity; Baskin and Baskin 2014b; Grime et al. 1981; Milberg et al. 2000; Morgan 1998; Plummer and Bell 1995; Scott and Morgan 2012), presumably as an adaptive strategy to prevent germination when seeds are buried deep in soil or under dense leaf litter where seed energy reserves are insufficient for successful emergence. On the other hand, in arid and semi-arid environments, increased germination in the dark is frequently reported (i.e., negative photoblasticity; Miranda-Jácome et al. 2013; Schütz et al. 2002; Thanos et al. 1991) and may be important to ensure that germination occurs in microsites where harsh solar irradiation is attenuated by nurse plant coverage, or to prevent germination on a rapidly drying or impenetrable soil surface (Bell et al. 1995). Finally, seeds may be insensitive to light availability (neutral photoblastic; Flores et al. 2015) including, in particular, larger seeds which may be sufficiently well reserved to successfully germinate in a range of microsite conditions (Carta et al. 2017). Differential photoblastic responses across species within a single ecological community are known to occur and may play a role in promoting spatial niche separation (Scott and Morgan 2012). Investigating variation in seed sensitivity to light amongst species in different ecological communities can improve our understanding of how light availability influences seed bank dynamics which, in turn, may have important consequences for our mechanistic understanding of species coexistence in spatially varying environments (Chesson 2000a).

Species’ seed germination responses may also be dependent on the microsite conditions individuals experience after dispersal (i.e., seed bank conditions). Many winter annual plant species are physiologically dormant after shedding from the mother plant and cannot germinate until dormancy is alleviated (Baskin and Baskin 2014a). Seed dormancy alleviation is influenced by abiotic conditions such as temperature and moisture availability (Schütz et al. 2002), which may vary even across local scales and thus influence spatial variation in dormancy depth within seed populations (Dwyer and Erickson 2016; Kaur et al. 2020). This may, in turn, lead to more-nuanced post-emergence site selection by species if the microsite conditions that most effectively alleviate dormancy are decoupled from those that promote germination.

In this study, we test the light requirements for seed germination under laboratory conditions for 12 common annual plant species occurring in the understory of York gum-jam woodlands in southwest Western Australia. This woodland is characterized by a sparse overstorey of *Eucalyptus loxophleba* (York gum) and *Acacia acuminata* (jam) which leads to natural variation in patch conditions such as the amount of shade and leaf litter as well as scattered woody debris. Thus, in addition to testing light sensitivity for germination, we also tested how variation in seed bank conditions influenced germination responses amongst species. In contrast to many studies stimulating variation in the seed bank environment under laboratory conditions, our study tested species’ germination after experiencing natural, field-based variation in seed bank conditions. As such, we assessed whether microsite conditions experienced in the seed bank in situ are associated with variation in: i) species’ overall germination proportion and ii) the magnitude of species’ germination responses to light relative to complete darkness.

## Methods

### Study system and species

Seeds of winter annual plants in the York gum-jam woodlands germinate following the onset of cool, wet conditions at the beginning of winter (June) and set seed in spring (October–November). Dispersed seeds spend the hot, dry summer–autumn period in the seed bank. Experimental work was conducted in the West Perenjori nature reserve (29° 28′ 40ʺ S, 116° 12′ 00ʺ E, close to the most northern extent of the York gum-jam woodlands (Fig. S1). Germination inducing rainfall in June averages 57 mm, and daily minimum and maximum temperatures in June average 7.6 ℃ and 19.3 ℃, respectively (Bureau of Meterology 2020). We selected 12 focal species which are common across the reserve, representing 5 families and 11 genera (Table 1). In addition, we chose these focal species because they exhibited considerable variation in their seed mass which, although not central to the focus of our study, allowed us to evaluate the relationship between seed mass and photoblasticity in this system.

**Table 1:** Intercept and regression coefficients from the germination ~ light treatment only models

Species	Family	Origin	Mean seed mass (mg)	Intercept	Light	RLG
*Arctotheca calendula* (L.) K.Lewin	Asteraceae	E	1.49	**– 0.90 (– 1.46, – 0.35)**	– 0.24 (– 0.68, 0.19)	0.46
*Daucus glochidiatus* (Labill.) Fisch., C.A.Mey. & Avé-Lall	Apiaceae	N	2.72	**2.80 (2.24, 3.49)**	**0.86 (0.03, 1.71)**	0.51
*Goodenia berardiana* (Gaudich.) Carolin	Goodeniaceae	N	2.47	**– 1.17 (– 1.71, – 0.67)**	**5.68 (4.79, 6.76)**	0.81
*Hyalosperma glutinosum* Steetz subsp. Glutinosum	Asteraceae	N	0.91	0.52 (– 0.16, 1.20)	0.70 (– 0.12, 1.56)	0.55
*Hypochaeris glabra* L	Asteraceae	E	0.42	**3.34 (2.62, 4.25)**	0.60 (– 0.36, 1.64)	0.50
*Lawrencella rosea* Lindl	Asteraceae	N	1.39	**– 1.59 (– 2.15, – 1.06)**	**3.21 (2.66, 3.83)**	0.83
*Plantago debilis* R. Br	Plantaginaceae	N	0.62	0.67 (– 0.01, 1.40)	0.03 (– 0.81, 0.87)	0.50
*Podolepis aristata* Benth. subsp. aristata	Asteraceae	N	0.21	**– 1.47 (– 2.19, – 0.84)**	0.10 (– 0.81, 1.00)	0.52
*Schoenia cassiniana* (Gaudich.) Steetz	Asteraceae	N	3.00	**– 0.66 (– 1.07, – 0.26)**	**1.79 (1.30, 2.34)**	0.69
*Trachymene cyanopetala* (F.Muell.) Benth	Araliaceae	N	2.09	– 0.47 (– 1.09, 0.16)	0.10 (– 0.23, 0.45)	0.52
*Trachymene ornata* (Endl.) Druce	Araliaceae	N	1.80	**– 1.93 (– 2.368 – 1.54)**	**0.75 (0.28, 1.23)**	0.65
*Velleia rosea* S. Moore	Goodeniaceae	N	2.82	**– 1.69 (– 2.49, − 0.91)**	**5.14 (4.11, 6.36)**	0.86

Intercept corresponds to the probability of germination under dark conditions, while Light is change in probability under light conditions. Values are in logits where the brackets are the 95% credible interval of the posterior distribution. Bolded text indicates that the parameter estimate was considered to be significant (i.e., the credible interval did not bound zero). Species-level relative germination (RLG) is also provided where values above 0.5 indicate increased germination under light and values below 0.5 indicated decreased germination under light

### Field design

Seeds of each focal species were collected at maturity in early October 2018 from senescing mother plants across a variety of environmental patches in the reserve, and were combined to control for possible effects of local adaptation and maternal effects. To expose the collected seeds to natural conditions in the soil seed bank, at the end of the 2018 growing season (early–mid October), we buried 50 intact and filled seeds of each species in 20 different locations across the reserve. Burial locations (patches) were systematically selected to capture measurable variation in different aspects of the abiotic environment known to be strongly associated with annual plant species turnover in this system (i.e., canopy cover, litter cover, and the presence of coarse woody debris).

Prior to being buried, seeds were separated from chaff and placed into thin nylon mesh bags mixed with 20 mL of coarse sand to reduce seed-seed contact and associated risks of pathogen-related mortality (Van Mourik, Stomph & Murdoch 2005). One row of 12 filled bags (i.e., one per species) was buried in a 1 cm deep trench in each patch (240 bags total; 12 species × 20 patches) and covered with a thin layer of topsoil (i.e., 1–2 mm). Consequently, within each seed bag, seeds were buried to a depth ranging from 1 cm to just below the topsoil, representing the range of depths that seeds in another Mediterranean annual plant system were typically buried (Traba et al. 2004). If coarse woody debris was present, the row of seed bags was buried immediately adjacent and parallel to the debris to ensure that all species experience similar conditions. In patches where leaf litter was present, it was carefully removed from the area in which the seed bags were to be buried and replaced after the seed bags were in position.

In mid-May 2019 (7 months after burial), just prior to the onset of germination-inducing rainfall, seed bags were exhumed and immediately placed into paper bags before being transported to the laboratory at Kings Park and Botanic Garden, Perth, Western Australia. It is unlikely that seeds were imbibed when retrieved from the field, because there was extremely limited precipitation over the month prior to retrieval (0.6 mm monthly total dispersed over two precipitation events; Bureau of Meteorology 2020).

### Seed bank environmental conditions

Canopy cover was measured by taking an upwards-facing wide-angle digital photograph (GoPro Hero5) approximately 30 cm above the point where seed bags were buried in each patch. The percentage of overhead canopy cover was estimated from the digital photographs using ImageJ (Schneider et al. 2012). We processed only the northern half of digital photographs to account for the orientation of the daily path of the sun throughout the growing season. The presence of leaf litter was recorded as a binary variable where a “presence” was recorded if the entire row of seed bags was covered, such that little-to-no surface soil was visible. In such microsites, leaf litter had a typical dry density of ~ 500 g/m^2^ and ranged from between 0.5 and 1 cm deep. Similarly, the presence of coarse woody debris was recorded as a binary variable.

### Germination experiment

Prior to conducting the germination experiment, seed fill (a proxy for viability) for each species was assessed on seeds retrieved from each patch using X-ray examination (Faxitron MX-20 digital X-ray cabinet, Tuscon, Arizona, USA). Under X-ray, a filled viable seed appears uniformly white/grey in the imagery as they contain a healthy endosperm and embryo. Non-filled seeds tend to have clear abnormalities, fractures, and dark shading, and are deemed non-viable.

The germination test was conducting by randomly dividing the seeds for a given species and patch into two equal groups. In a few cases, some seeds were lost from the mesh bag prior to the start of the experiment, so that the total number of seeds across both groups was less than 50. There were no obvious signs that germination had occurred in any of the mesh bags prior to extraction from the field. Each group of seeds was placed on a sheet of filter paper moistened with 10 mL/L Plant Preservation Mixture (Plant Cell Technology, Washington D.C.) in a Petri-dish and wrapped in cling wrap to prevent moisture loss. One Petri-dish from each mesh bag was randomly assigned to the “dark” treatment and was wrapped in two layers of aluminium foil to completely prevent light penetration, while the other petri dish was assigned to the “light” treatment and was left unwrapped. Application of the dark treatment occurred immediately after the moistening of filter paper to avoid imbibition in the presence of light. Thus, in total, there were 20 paired replicates of the light/dark treatment for each species (i.e., 40 Petri dishes per species).

Petri dishes across both treatments were randomly assigned a position inside a single growth cabinet (Contherm 6400CP4, Contherm Scientific Ltd, New Zealand). Light was provided by cool white, fluorescent tubes (30 μmol m^−2^ s^−1^, 400–700 nm) with a 12/12 h day/night regime. The temperature regime inside the growth cabinet was a 12/12 h diurnal cycle of 19 °C (during the day) and 7 °C (during the night) to mimic average maximum and minimum daily temperatures during peak germination at Perenjori (Bureau of Meterology 2020). Germination in the light treatment was scored every 7 d, beginning 7 d after the experimental trial was conducted, for 35 d. During each inspection, seeds were recorded as germinated when the radicle emerged from the seed coat, and were removed after counting. It was not possible to inspect germination in the dark treatment through time, and so, germination was instead inspected only at the end of the 35-day period. All Petri dishes were re-randomized within the growth cabinet after each 7-day interval.

Overall, seed fill was high for all species (> 90%) and was mostly unresponsive to measured environmental variables (Table S1). Nevertheless, for the purposes of statistical analyses, we adjusted germination proportions for each species to account for seed fill by multiplying the total number of seeds in each Petri-dish by the species and patch-specific seed fill fractions obtained from X-ray analysis (rounding up to the next whole number). In addition, individuals that had broken their seed coat but either had no radicle or an unhealthy-appearing radicle were considered non-viable and were subtracted from the total number of viable seeds in each Petri-dish. In a small proportion of the cases where germination was close to 100%, the seed fill-adjusted total number of seeds was lower than the number of germinating seeds due to rounding and underestimation of seed fill using X-ray analysis (30 out of 478 petri dishes). In these cases, the adjusted number of seeds was instead considered to be equal to the number of germinating seeds, although this typically represented a change in the seed fill-adjusted total number of seeds of only one seed and in a very small number of cases (*n* = 5), two seeds.

### Statistical analyses

All statistical analyses were conducted in R Version 4.0.3 (R Core Team 2020) via RStudio Version 1.3.1093 (RStudio Team 2020). We used a Bayesian framework for statistical modelling. Bayesian models were fitted in Stan (Carpenter et al. 2017), via the *brms* package in R (Bürkner 2017). Four MCMC chains were used in all models with a minimum of 3000 iterations and a burn-in of 1500 iterations. Because we used the default, weakly informative prior distributions when estimating regression coefficients and variance parameters in *brms*, the central tendencies of posterior distributions are analogous to the parameter estimates obtained from generalized linear models using frequentist methods. We assessed the R^ statistic to evaluate model convergence where *R*^ < 1.1 was considered to indicate adequate model convergence (Gelman and Rubin 1992). Parameter estimates were interpreted as being “significant” when the 95% credible interval of the posterior distribution did not bound zero.

To first assess the overall effect of the light treatment on germination, mixed-effects binomial models were used to assess the probability of a seed germinating in either light or dark conditions for each species according to the following equation (i.e., the “light treatment only” model):

$${\text{Probability}} \,{\text{of}}\,{\text{ germination}}\, \left( {n\, = \, {\text{number}}\,{\text{of}}\, {\text{successful}}\,{\text{ germinants}}\,,\, p\, = \,{\text{number}}\,{\text{of}}\,{\text{viable}}\, {\text{seeds}}} \right) \sim a + b_{1} \, \times \,{\text{light }}\,{\text{treatment}}\, + \,\left( {1\,\left| {{\text{patch}}/{\text{petri }}\,{\text{dish}}} \right.} \right),$$

where *a* denotes the estimated global intercept and *b* denotes the estimated slope value. Patch was included as a random intercept. In addition, petri dish was included as a nested observation-level random effect (OLRE) to account for overdispersion potentially emerging as a consequence of the aggregation of individual seed responses within each trial (Elston et al. 2001). To confirm that the inclusion of Petri-dish improved model performance, we fit a model excluding the OLRE for each species and compared it to the full model using expected log predictive density, which was estimated using leave-one-out cross validation (Vehtari et al. 2018). For 11 out of the 12 species, the model including the OLRE had a higher ELPD, indicating a better fit, and in five of these cases, the difference in ELPD was significant, suggesting that the OLRE was capturing significant observation-level variation (Table S2). We therefore retained Petri-dish as a random effect in all binomial models.

Relative light-requirement for germination (RLG) was then expressed for each species, *s*, using the point estimates from the posterior distribution of the estimated parameters for probability of germination in the light, *G*_light_ and in the dark, *G*_dark_

$${\mathrm{RLG}}_{s}= {G}_{\mathrm{light}} / ({G}_{\mathrm{light}}+ {G}_{\mathrm{dark}}),$$

where values of RLG greater than 0.5 indicate greater germination in the light than in the dark and vice versa for values of RLG lower than 0.5 (Milberg et al. 2000).

To assess the effect of patch conditions on species’ germination responses, we first tested how species’ overall probability of germination varied across patches (i.e., the “light + seed bank” model). For a given species, germination responses across all Petri dishes (i.e., seeds under both light and dark conditions) were regressed as a function of the measured environmental variables using mixed-effects binomial models, including patch and Petri-dish as random intercepts

$${\text{Probability}}\,{\text{ of}}\,{\text{ germination}}\, \left( {n\, = \,{\text{number}}\,{\text{ of}}\,{\text{ successful}}\, {\text{germinants}}\,,\, p\, = \,{\text{number}}\,{\text{ of}}\, {\text{viable}}\,{\text{ seeds}}} \right) \sim a + b_{1} \times {\text{light}}\,{\text{ treatment}}\, + \,b_{2} \times {\text{sqrt}}\left( {{\text{canopy}}\,{\text{ cover}}} \right)\, + \,b_{3} \times {\text{CWD}}\, + \,\left( {1\,{\text{|patch}}/{\text{petri}}\,{\text{ dish}}} \right).$$

The environmental variables included in these models were the percentage of overstory canopy cover and the presence of coarse woody debris. Overstory canopy cover and the presence of leaf litter on the seed bags were strongly and positively correlated, so we chose to omit the presence of leaf litter to improve the interpretability of models. Canopy cover was right-skewed and was therefore sqrt-transformed prior to analyses. The light/dark treatment was also included as a variable in these models to account for the significant bimodality of the germination responses for photoblastic species.

The effect of seed bank conditions on species’ germination response to light was then assessed by regressing the probability of germination across all Petri dishes as a function of the light/dark treatment, each environmental variable and their two-way interaction according to the following equation (i.e. the “light * seed bank” model):

$${\text{Probability}}\,{\text{ of}}\,{\text{ germination}}\, \left( {n\, = \,{\text{number}}\,{\text{ of}}\,{\text{ successful}}\, {\text{germinants}}\,,\, p\, = \,{\text{number}}\,{\text{ of}}\, {\text{viable}}\,{\text{ seeds}}} \right) \sim a + b_{1} \times {\text{light}}\,{\text{ treatment}}\, + \,b_{2} \times {\text{sqrt}}\left( {{\text{canopy}}\,{\text{ cover}}} \right)\, + \,b_{3} \times {\text{CWD}}\,{ + }\,b_{4} \times {\text{light treatment}}\, \times \,{\text{sqrt}}\left( {{\text{canopy}}\,{\text{ cover}}} \right)\,{ + }\,b_{5} \times {\text{light treatment}}\, \times \,{\text{CWD}}\, + \,\left( {1\,{\text{|patch}}/{\text{petri}}\,{\text{ dish}}} \right).$$

Again, a mixed-effects binomial model was used, including patch and Petri-dish as nested random intercepts. In cases where a statistical interaction was significant, we used the *emtrends* and *emmeans* functions in the *emmeans* package (Lenth et al. 2020) to estimate the marginal mean slope of the effect of the environmental variable on the probability of germination under light and dark conditions, separately.

## Results

Mean seed fill across all patches ranged from 91% (*Arctotheca calendula*) to 97% (*Trachymene ornata*; Table S1). The median percentage of germinating seeds ranged from 12.8% (*T. ornata*) to 93.5% (*Daucus glochidiatus*) under dark conditions and 15.5% (*Podolepis aristata*) to 100% (*Hypochaeris glabra*) under light conditions (Table S3).

### Species overall germination responses to light vs. complete darkness

**Fig. 1:**
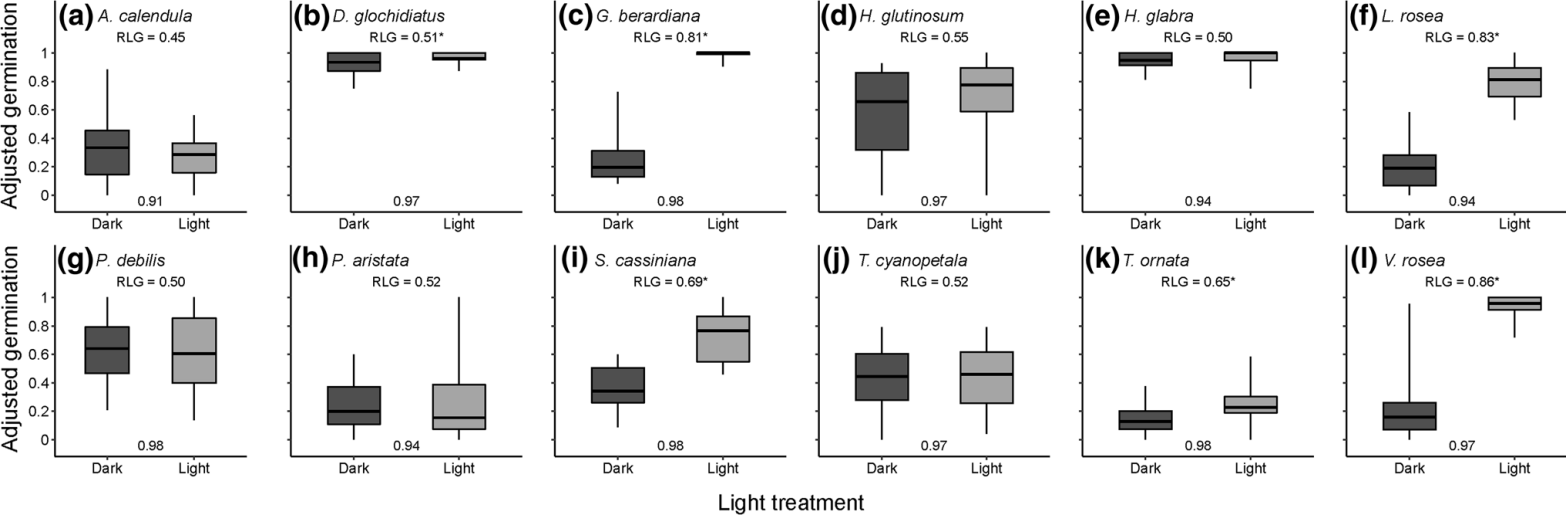
Adjusted germination percentages for 12 focal species in light (12 h diurnal light) or dark conditions. Boxes represent the interquartile range and median and the whiskers represent the range. Significant differences in the probability of germination between light and dark conditions in the binomial mixed-effects model are represented by *. Species-level median fill rates are presented in grey at the bottom of each panel

Six out of the 12 species exhibited significant germination responses to the light treatment, and in all these cases, they were positive (Fig. 1). However, the relative magnitudes of these significant responses were highly variable, with RLG ranging between 0.51 (*D. glochidiatus*) and 0.86 (*Velleia rosea;* Table 1). Only one species, *A. calendula,* exhibited a negative response to light, and even in this case, the effect was not significant. Germination was observed under light and dark conditions for all species.

### Seed bank microsite conditions and species’ overall germination

**Fig. 2:**
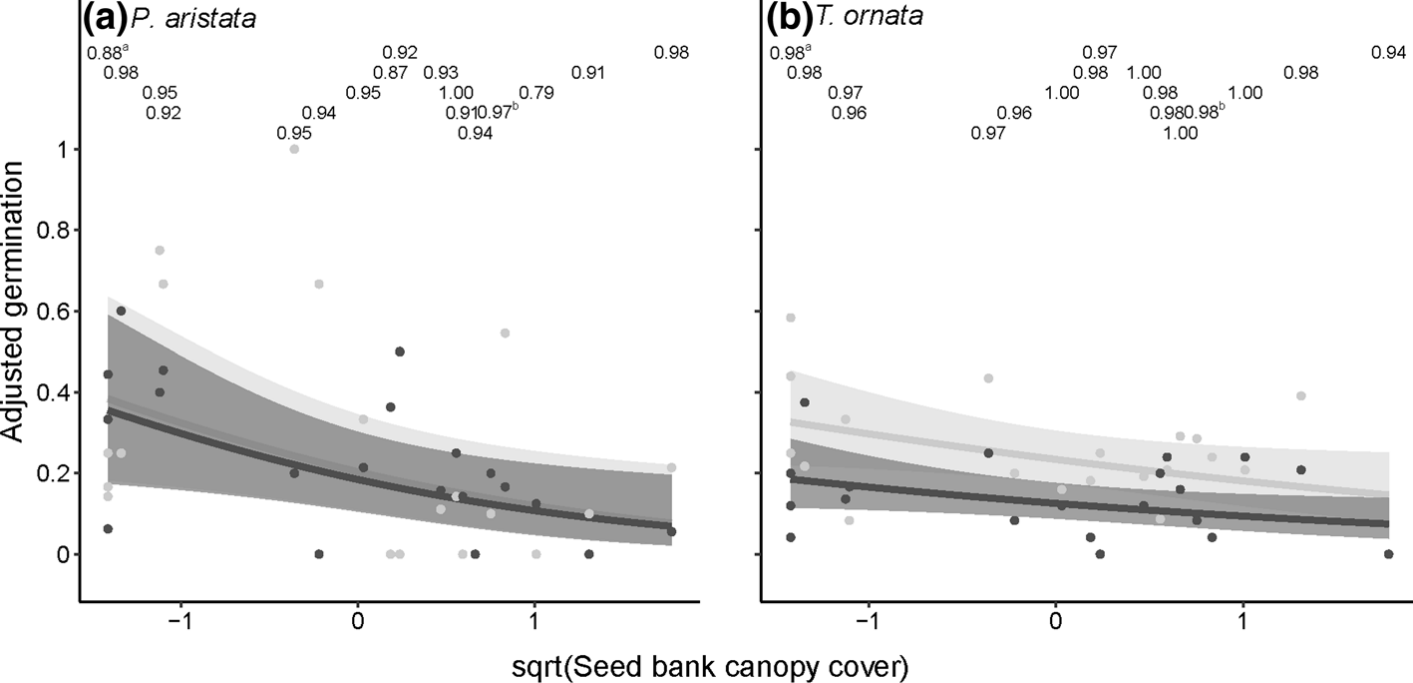
Adjusted germination percentages for two focal species, a) *P. aristata* and b) *T. ornata,* retrieved from patches across a gradient of canopy cover (scaled square root) in light (12 h diurnal light, grey points) or dark conditions (black points). Solid lines represent the fitted relationships from the “main effects only” model while the grey fields represent the 95% credible interval around the fitted relationship. Patch-level seed fill rates are presented in grey at the top of the panel. ^a^ and ^b^ indicates that the noted fill rate is instead the mean fill rate for canopy cover values of zero (-1.41 on the scale of the x-axis) and canopy cover values of ~ 34% and 36% (0.75 and 0.83 on the scale of the x-axis), respectively, to improve visual clarity

Of the two environmental variables measured in the seed bank locations, species’ overall germination (i.e., across light and dark conditions) responded most strongly to the amount of overstory canopy cover, where *P. aristata* and *T. ornata* had significantly lower germination as the amount of canopy cover increased (Table 2; Fig. 2). The presence of coarse woody debris had no effect on germination for any species.

**Table 2:** Intercept and regression coefficients from the germination ~ light + seed bank models

Species	Intercept	Light	sqrt(Canopy cover)	CWD
*Arctotheca calendula*	**– 0.87 (– 1.64, – 0.12)**	– 0.24 (– 0.69, 0.22)	0.28 (– 0.29, 0.85)	– 0.08 (– 1.12, 1.05)
*Daucus glochidiatus*	**3.01 (2.18, 4.02)**	**0.86 (0.01, 1.70)**	– 0.05 (– 0.59, 0.47)	– 0.30 (– 1.45, 0.79)
*Goodenia berardiana*	**– 0.70 (– 1.43, – 0.03)**	**5.70 (4.82, 6.73)**	– 0.10 (– 0.60, 0.43)	– 0.96 (– 1.96, 0.04)
*Hyalosperma glutinosum*	0.50 (– 0.42 1.44)	0.70 (– 0.16, 1.56)	– 0.17 (– 0.82, 0.51)	0.05 (– 1.21, 1.33)
*Hypochaeris glabra*	**3.27 (2.22, 4.52)**	0.63 (– 0.34, 1.72)	– 0.19 (– 0.98, 0.58)	0.33 (– 1.17, 1.76)
*Lawrencella rosea*	**– 2.03 (– 2.85, – 1.28)**	**3.24 (2.67, 3.91)**	– 0.05 (– 0.55, 0.47)	0.79 (– 0.22, 1.80)
*Plantago debilis*	0.35 (– 0.59, 1.31)	0.04 (– 0.81, 0.93)	0.41 (– 0.22, 1.03)	0.63 (– 0.60, 1.91)
*Podolepis aristata*	**– 1.54 (– 2.44, – 0.74)**	0.12 (– 0.77, 0.99)	**– 0.63 (– 1.22, – 0.11)**	0.10 (– 0.92, 1.16)
*Schoenia cassiniana*	**– 0.83 (– 1.37, – 0.30)**	**1.80 (1.26, 2.36)**	0.08 (– 0.27, 0.43)	0.32 (– 0.37, 1.00)
*Trachymene cyanopetala*	– 0.11 (– 0.95, 0.74)	0.11 (– 0.24, 0.46)	– 0.31 (– 0.94, 0.32)	– 0.72 (– 1.95, 0.46)
*Trachymene ornata*	**– 1.80 (– 2.32, – 1.31)**	**0.76 (0.30, 1.25)**	**– 0.32 (– 0.62, – 0.02)**	– 0.29 (– 0.87, 0.34)
*Velleia rosea*	**– 1.71 (– 2.71, – 0.74)**	**5.22 (4.19, 6.46)**	– 0.65 (– 1.32, 0.00)	0.03 (– 1.29, 1.37)

Values in brackets are the 95% credible interval of the posterior distribution. Bolded text indicates that the parameter estimate was considered to be significant (i.e., the credible interval did not bound zero). Values are in logits and are in standardised units for scaled sqrt(Canopy cover). CWD indicates the effect of the presence of coarse woody debris

### Seed bank microsite conditions and species’ germination responses to light vs. complete darkness

There was only one statistically significant interaction between the effect of the light treatment and the measured seed bank conditions out of the 12 focal species studied (Table S4). This interaction indicated that the probability of germination for *Goodenia berardiana* declined with canopy cover under dark germination conditions, but increased with canopy cover under light germination conditions (Figure S2). However, separately inspecting the slopes of the marginal trends of germination probability with canopy cover under light or dark conditions for *G. berardiana* revealed that they were not significantly different from zero under either incubation condition (slope value and credible interval under light in logits = 0.99 [− 0.13, 2.08], slope value and credible interval under dark in logits = − 0.25 [− 0.76, 0.26]).

## Discussion

We report the germination response of 12 winter annual species from a semi-arid woodland in the context of variation in seed bank conditions as dictated by differing canopy cover, leaf litter, and scattered woody debris. Seed populations of half of the species exhibited significant, positive responses to exposure to light in their germination fractions, although the magnitude of these responses varied considerably between species. Spatial variation in canopy cover above the seed bed was found to influence germinability for two species; although only seeds of one of these species, *T. ornata*, responded significantly in its germination to both the amount of canopy cover and light treatment. There was only minor evidence to suggest that seed bank conditions may also influence species’ relative responses to light and dark conditions.

### Light requirements for germination

Six out of the 12 focal species exhibited significant positive germination responses to light, suggesting that positive photoblasticity is a relatively common phenomenon in our study system. This finding is consistent with a number of other studies (Bunker 1994; Plummer and Bell 1995) in Australian annual plant systems including Scott and Morgan (2012) who found that 15 out of 19 species of forbs from a south eastern temperate grassland had higher germination under light than continuous darkness. In contrast to a number of other studies in semi-arid and Mediterranean environments (Miranda-Jácome et al. 2013; Schütz et al. 2002; Thanos et al. 1991); however, we found very little evidence for negative photoblasticity (i.e., higher germination in the dark). Schütz et al. (2002) hypothesized that light-inhibited germination should be more common in Mediterranean ecosystems where germination on the soil surface is risky due to rapid loss of surface soil moisture. Indeed, in support of this hypothesis, Carta et al. (2017) showed in a global synthesis of species’ germination behaviour that species exhibiting negative photoblasticity occur most frequently in open, seasonal, or arid ecosystems like the York gum woodlands. Nevertheless, Carta et al. (2017) also estimated that only 4% of taxa in these regions are likely to exhibit strongly photoinhibited germination. Thus, the fact that we did not observe significant negative photoblasticity may simply reflect the number of focal species that we investigated.

Seed mass is hypothesized to have coevolved with species’ germination requirement for light as an adaptive strategy to ensure that smaller seeds, which have less maternal provisioning, germinate when close to the soil surface or in open microsites. For example, species with smaller seeds have been shown to be more likely to exhibit positive photoblasticity in both observational studies and global syntheses (Carta et al. 2017; Grime et al. 1981; Milberg et al. 2000). Interestingly, after conducting further regression analysis, we found that larger seeded species instead tended to exhibit stronger, positive germination responses to light than smaller seeded species, although this relationship was weak (Fig. S3; Table S5). Given the relatively small number of focal species and plant families that we studied, and the fact that we did not observe species with significantly higher germination under dark conditions, it is not possible to infer whether this pattern is more broadly applicable to our, or other, systems. Nevertheless, it does suggest that the adaptive value of strong light requirements for germination may extend beyond ensuring that individuals have sufficient seed resources to successfully emerge in our system and could be further investigated by assessing the relationship between photoblasticity and traits relevant to later life stages (Donohue et al. 2010). For example, it may be that sensitivity to light operates as a gap-detecting mechanism, which promotes germination in open or bare microsites and would be selected for if species possessed traits that confer a high probability of post-establishment success in these microsites. It is also possible that seeds depend on other environmental cues in addition to or instead of light to indicate depth of burial. For example, in Mediterranean annuals, Saatkamp et al. (2011) showed that seeds of some species require diurnal fluctuations in temperature to germinate and proposed that this may have adaptive value as a depth-sensing mechanism, because the magnitude of diurnal fluctuations in temperature decrease with depth. Exploring the relationship between seed size and the interactive effect of sensitivity to fluctuations in germination temperature and light would therefore be of significant value in this system.

Seed germination responses to light conditions can be complex and are known to vary with seed dormancy status (Derkx and Karssen 1993) and other seasonally varying environmental factors including temperature and the chemical environment (Karlsson and Milberg 2007; Merritt et al. 2006). It is therefore important to acknowledge that our germination assay captures only a “snapshot” of the possible phenotypic variation in light/dark response of the retrieved seeds. Nevertheless, the temperature regime in the growth chamber was selected to simulate the climatic conditions experienced by seeds in a typical early winter (beginning of the growing season) in the northern extent of the York gum-jam woodlands and as known as conducive to germination of annuals from this region (Merritt et al. 2006). In addition, seeds were permitted to over-summer in the field to promote dormancy loss via after-ripening (Schütz et al. 2002). Thus, observed photoblastic responses are most likely consistent with “average” patterns in the field.

### Seed bank conditions

In our study, overall germination (i.e., across the light/dark treatment) varied most strongly in response to seed bank overstory cover for two species. This effect was consistent across species where germinability decreased for both *P. aristata* and *T. ornata* as canopy cover increased. Previous studies of seeds with physical dormancy have shown that germination fractions are higher when seeds are retrieved from open microsites compared to shaded microsites, most likely due to higher rates of seed coat softening (seed permeability) in open microsites (Jaganathan 2018; Rice 1985; Vázquez-Yanes and Orozco-Segovia 1982). However, we are aware of few studies in other systems (but see Dwyer and Erickson 2016) investigating the effect of in situ burial under canopy cover for seeds of species likely to exhibit physiological dormancy (see Hidayati et al. 2019; Hoyle et al. 2008; Schütz et al. 2002). In our system, canopy cover is known to reduce the maximum temperature at the soil surface as well as the amplitude of diurnal temperature fluctuations (Dwyer and Erickson 2016). Our findings for *P. aristata* and *T. ornata* are therefore consistent with many other studies, which find that warmer temperatures both in situ and in vitro tend to alleviate dormancy in annual plants with physiological dormancy (Dwyer and Erickson 2016; Schütz et al. 2002). Interestingly, the presence of coarse woody debris, which is also known in other systems to reduce temperature fluctuations, reduce the rate of soil moisture loss and influence the soil chemical environment (Goldin and Hutchinson 2013, 2014; Gray and Spies 1997), did not exert strong effects on overall germinability in our species, despite the fact that these environmental factors are well known to influence the alleviation of dormancy in other ephemeral species (Baker et al. 2005; Merritt et al. 2006; Schütz et al. 2002).

Seed germination response to light is known to depend in some cases on other presiding environmental factors prior, during, and following imbibition (see Pons 2013 and studies cited within). Ultimately, we found little evidence to suggest that seed bank conditions influence the relative light requirement for seed germination, except in the case of *G. berardiana.* For this species, germination in complete darkness showed a weak negative trend with increasing seed bank canopy cover, whereas germination in the light treatment was very high regardless of the seed bank conditions. However, post hoc analysis of the effect of canopy cover for the light and dark data separately revealed that neither slope was significant, suggesting that the difference between the slopes was relatively minor. Few studies, if any, have addressed how variation in the seed bank conditions, which we chose to measure, influences species’ germination response to light, so it is unclear whether our findings are consistent with those from other systems. For laboratory-stored seeds of some southwest Western Australian annuals, Schütz et al. (2002) found that the relative proportion of seeds germinating under light or dark conditions changed for *Podotheca gnaphalioides* depending on the temperature of dry storage. However, the temperature of storage in this case was between 5 °C and 25 °C, which likely captures a wider, and cooler, range of temperatures than what seeds would have experienced in the seed bank. Thus, it may be in our study that we captured insufficient variation in important environmental conditions across the different patches to induce changes in the light sensitivity of seeds.

### Ecological consequences of species’ seed germination response

Species-specific germination strategies may promote coexistence if it causes species to partition their niches across space. In our case, we observed at least two germination syndromes in response to light, being either neutral or positive photoblasticity. Amongst the positive photoblastic responses, however, species appeared to be differentiated depending on the magnitude of their response. For example, species such as *G. berardiana, L. rosea*, and *V. rosea* exhibited an “all-or-nothing” response to light with correspondingly high RLGs (i.e., > 0.80). In contrast, the effect of light on the remaining positively photoblastic species was substantially more reserved, especially in the case of *D. glochidiatus* which had an RLG of just 0.51. Taken as a whole, these results suggest that niche separation across spatial gradients of light availability may occur not only through the direction of seed photoblasticity but also through its magnitude.

In another study in our system, Dwyer and Erickson (2016) found that the germination of four species, including two species studied here, was a function of both the region (i.e., warm or cool) where seeds were buried throughout the summer months as well as the germination temperature, thereby showing that temperature can mediate cumulative germination fractions through its influence on multiple stages of seed life history*.* In a similar way, our finding that germination proportions varied across a gradient of canopy cover independently of the light treatment in the laboratory points to a complex effect of the spatial environment whereby germination proportions were determined not only by seed bank conditions coinciding with the arrival of germination-inducing rainfall (i.e., the light treatment) but also with those occurring throughout the summer (Kaur et al. 2020). Ultimately, this phenomenon may facilitate more nuanced site selection by species, promoting coexistence through niche separation, although this will require further investigation.

## Supplementary Information

Below is the link to the electronic supplementary material.

Supplementary file1 (DOCX 2938 KB)

## Data Availability

Data is available on the Dryad database (doi: 10.5061/dryad.gxd2547nx).

## Code availability

R code used to conduct the statistical analyses is available on the Dryad database (doi: 10.5061/dryad.gxd2547nx).
