# Supplementary material for: Variable seed bed microsite conditions and light influence germination in Australian winter annuals
Source: Oecologia. 2022 Jan 9;198(4):865–75. doi: 10.1007/s00442-021-05091-7 (PMC9056468; doi:10.1007/s00442-021-05091-7)
Supplement: Supplementary file 1 — Supplementary file1 (DOCX 2938 KB) [file 442_2021_5091_MOESM1_ESM.docx]

| Table S1: Intercept and regression coefficients from the probability of seed fill ~ environment models. Values in brackets are the 95% credible interval of the posterior distribution. Bolded text indicates that the parameter estimate was considered to be significant (i.e. the credible interval did not bound zero). Values are in logits and are in standardised units for sqrt(Canopy cover). Median proportion of filled seeds is also provided where the values in brackets are the lower and upper 95% quantiles. | | | | | |
| --- | --- | --- | --- | --- | --- |
| Species | Intercept | sqrt(Canopy cover) | CWD | Median seed fill |  |
| *Arctotheca calendula* | **2.31 (1.99, 2.67)** | -0.04 (-0.31, 0.22) | 0.02 (-0.48, 0.50) | 0.91 (0.86, 0.96) |  |
| *Daucus glochidiatus* | **3.79 (3.23, 4.47)** | -0.10 (-0.53, 0.29) | -0.73 (-1.59, 0.06) | 0.97 (0.94, 1) |  |
| *Goodenia berardiana* | **3.35 (2.76, 4.05)** | 0.06 (-0.41, 0.55) | 0.42 (-0.52, 1.45) | 0.98 (0.91, 1) |  |
| *Hyalosperma glutinosum* | **3.51 (2.90, 4.19)** | 0.06 (-0.41, 0.53) | -0.15 (-1.06, 0.81) | 0.97 (0.91, 1) |  |
| *Hypochaeris glabra* | **2.69 (1.90, 3.59)** | 0.13 (-0.47, 0.74) | 0.31 (-0.97, 1.49) | 0.94 (0.8, 1) |  |
| *Lawrencella rosea* | **2.85 (2.38, 3.35)** | -0.02 (-0.36, 0.33) | -0.08 (-0.74, 0.61) | 0.94 (0.88, 0.98) |  |
| *Plantago debilis* | **3.92 (3.27, 4.70)** | -0.41 (-0.99, 0.11) | -0.05 (-1.04, 0.92) | 0.98 (0.95, 1) |  |
| *Podolepis aristata* | **2.44 (1.92, 3.06)** | 0.16 (-0.25, 0.57) | 0.51 (-0.35, 1.32) | 0.94 (0.79, 0.98) |  |
| *Schoenia cassiniana* | **3.91 (3.26, 4.67)** | 0.02 (-0.48, 0.52) | -0.26 (-1.23, 0.67) | 0.98 (0.96, 1) |  |
| *Trachymene cyanopetala* | **3.38 (2.74, 4.16)** | 0.13 (-0.39, 0.65) | 0.41 (-0.57, 1.48) | 0.97 (0.92, 1) |  |
| *Trachymene ornata* | **3.88 (3.17, 4.70)** | -0.02 (-0.57, 0.52) | 0.09 (-1.00, 1.19) | 0.98 (0.95, 1) |  |
| *Velleia rosea* | **3.27 (2.62, 4.08)** | 0.18 (-0.35, 0.59) | 0.26 (-0.79, 1.32) | 0.97 (0.88, 1) |  |

| Table S2: Difference in expected log predictive density (ELPD) estimated using leave-one-out cross validation for the “light treatment only” model including (OLRE) or not including (No OLRE) an observation-level random effect. Model are ordered by performance where models with less negative values perform better. Differences in performance between models are considered significant and marked in bold when the difference in ELPD (ΔELPD) is greater than twice the standard error of the difference in ELPD (ΔELPD_SE) | | | | |
| --- | --- | --- | --- | --- |
| Species | Model | ΔELPD | ΔELPD_SE | ELPD |
| *Arctotheca calendula* | No OLRE | 0.0 | 0.0 | -105.2 |
|  | OLRE | -0.3 | 2.5 | -105.5 |
|  |  |  |  |  |
| *Daucus glochidiatus* | OLRE | 0.0 | 0.0 | -62.2 |
|  | No OLRE | -2.4 | 1.4 | -64.6 |
|  |  |  |  |  |
| *Goodenia berardiana* | OLRE | 0.0 | 0.0 | -68.9 |
|  | No OLRE | -0.9 | 1.1 | -69.8 |
|  |  |  |  |  |
| *Hyalosperma glutinosum* | **OLRE** | **0.0** | **0.0** | **-89.1** |
|  | No OLRE | -19.1 | 7.7 | -108.2 |
|  |  |  |  |  |
| *Hypochaeris glabra* | OLRE | 0.0 | 0.0 | -48.4 |
|  | No OLRE | -0.1 | 0.9 | -48.5 |
|  |  |  |  |  |
| *Lawrencella rosea* | OLRE | 0.0 | 0.0 | -87.5 |
|  | No OLRE | -2.5 | 1.7 | -90.0 |
|  |  |  |  |  |
| *Plantago debilis* | **OLRE** | **0.0** | **0.0** | **-107.3** |
|  | No OLRE | -56.2 | 15.8 | -163.4 |
|  |  |  |  |  |
| *Podolepis aristata* | **OLRE** | **0.0** | **0.0** | **-78.5** |
|  | No OLRE | -8.8 | 4.3 | -87.3 |
|  |  |  |  |  |
| *Schoenia cassiniana* | **OLRE** | **0.0** | **0.0** | **-104.5** |
|  | No OLRE | -15.2 | 5.5 | -119.7 |
|  |  |  |  |  |
| *Trachymene cyanopetala* | OLRE | 0.0 | 0.0 | -102.7 |
|  | No OLRE | -0.5 | 1.2 | -103.2 |
| *Trachymene ornata* | OLRE | 0.0 | 0.0 | -98.0 |
|  | No OLRE | -4.0 | 3.0 | -102.0 |
|  |  |  |  |  |
| *Velleia rosea* | **OLRE** | **0.0** | **0.0** | **-83.6** |
|  | No OLRE | -18.0 | 6.3 | -101.6 |

| Table S3: Median germination percentages for each focal species under dark and diurnal light (12h / 12h) germination conditions | | |  |
| --- | --- | --- | --- |
|  | Median germination | | |
| Species | Dark | Light | |
| *Arctotheca calendula* | 33.4% | 28.6% | |
| *Daucus glochidiatus* | 93.5% | 96.2% | |
| *Goodenia berardiana* | 19.5% | 100.0% | |
| *Hyalosperma glutinosum* | 65.8% | 77.5% | |
| *Hypochaeris glabra* | 95.0% | 100.00% | |
| *Lawrencella rosea* | 19.0% | 81.2% | |
| *Plantago debilis* | 64.1% | 60.5% | |
| *Podolepis aristata* | 20.0% | 15.5% | |
| *Schoenia cassiniana* | 34.1% | 76.6% | |
| *Trachymene cyanopetala* | 44.5% | 45.8% | |
| *Trachymene ornata* | 12.8% | 22.9% | |
| *Velleia rosea* | 16.0% | 95.8% | |

| Table S4: Intercept and regression coefficients from the probability of germination ~ light * environment models. Values in brackets are the 95% credible interval of the posterior distribution. Bolded text indicates that the parameter estimate was considered to be significant (i.e. the credible interval did not bound zero). Values are in logits and are in standardised units for sqrt(Canopy cover). | | | | | | |
| --- | --- | --- | --- | --- | --- | --- |
| Species | Intercept | Light | sqrt(Canopy cover) | CWD | Light*sqrt(Canopy cover) | Light*CWD |
| *Arctotheca calendula* | **-0.88 (-1.73, -0.08)** | -0.24 (-0.90, 0.43) | 0.46 (-0.14, 1.12) | -0.08 (-1.25, 1.12) | -0.35 (-0.87, 0.15) | 0.01 (-0.92, 0.95) |
| *Daucus glochidiatus* | **3.12 (2.22, 4.26)** | 0.77 (-0.58, 2.09) | -0.26 (-0.96, 0.40) | -0.41 (-1.80, 0.94) | 0.53 (-0.39, 1.50) | 0.25 (-1.63, 2.14) |
| *Goodenia berardiana* | -0.65 (-1.38, 0.04) | **5.61 (4.28, 7.3)** | -0.25 (-0.78, 0.25) | **-1.08 (-2.13, -0.05)** | **1.24 (0.19, 2.39)** | 0.92 (-1.06, 2.77) |
| *Hyalosperma glutinosum* | 0.51 (-0.58, 1.59) | 0.69 (-0.57, 1.97) | -0.33 (-1.12, 0.46) | 0.01 (-1.57, 1.56) | 0.37 (-0.58, 1.35) | 0.07 (-1.75, 1.93) |
| *Hypochaeris glabra* | **3.49 (2.37, 4.87)** | 0.38 (-1.02, 2.04) | -0.07 (-1.02, 0.88) | -0.04 (-1.84, 1.74) | -0.34 (-1.65, 0.90) | 0.94 (-1.41, 3.29) |
| *Lawrencella rosea* | **-2.12 (-3.06, -1.29)** | **3.39 (2.51, 4.45)** | -0.09 (-0.73, 0.53) | 0.90 (-0.22, 2.11) | 0.06 (-0.57, 0.72) | -0.19 (-1.47, 1.10) |
| *Plantago debilis* | 0.16 (-0.91, 1.24) | 0.47 (-0.8, 1.73) | 0.20 (-0.60, 0.98) | 1.06 (-0.47, 2.62) | 0.47 (-0.48, 1.44) | -0.87 (-2.71, 0.97) |
| *Podolepis aristata* | **-1.22 (-2.24, -0.31)** | -0.61 (-1.98, 0.72) | **-0.75 (-1.47, -0.07)** | -0.51 (-1.81, 0.85) | 0.26 (-0.70, 1.21) | 1.35 (-0.45, 3.16) |
| *Schoenia cassiniana* | **-0.85 (-1.49, -0.24)** | **1.86 (1.06, 2.73)** | 0.1 (-0.35, 0.57) | 0.37 (-0.54, 1.29) | -0.05 (-0.64, 0.54) | -0.09 (-1.29, 1.13) |
| *Trachymene cyanopetala* | 0.02 (-0.87, 0.87) | -0.13 (-0.62, 0.37) | -0.22 (-0.86, 0.46) | -0.97 (-2.20, 0.29) | -0.17 (-0.55, 0.21) | 0.46 (-0.24, 1.20) |
| *Trachymene ornata* | **-1.70 (-2.28, -1.16)** | 0.57 (-0.11, 1.27) | -0.28 (-0.70, 0.12) | -0.51 (-1.31, 0.33) | -0.08 (-0.60, 0.44) | 0.39 (-0.65, 1.41) |
| *Velleia rosea* | **-1.63 (-2.85, -0.44)** | **5.05 (3.44, 6.81)** | -0.69 (-1.58, 0.18) | -0.17 (-1.88, 1.55) | 0.06 (-1.15, 1.31) | 0.54 (-1.79, 2.94) |

| Table S5: Intercept and regression coefficients from the linear Bayesian model of relative light germination (RLG) ~ species-level mean seed mass. Variance in RLG explained by species-level mean seed mass is also included. Values in brackets are the 95% credible interval of the posterior distribution. | | |
| --- | --- | --- |
| Parameter | Estimate | R^2^ |
| Intercept | 0.49 (0.31, 0.69) | 0.26 (0.00, 0.54) |
| Mean seed mass | 0.08 (-0.03, 0.17) |  |

| 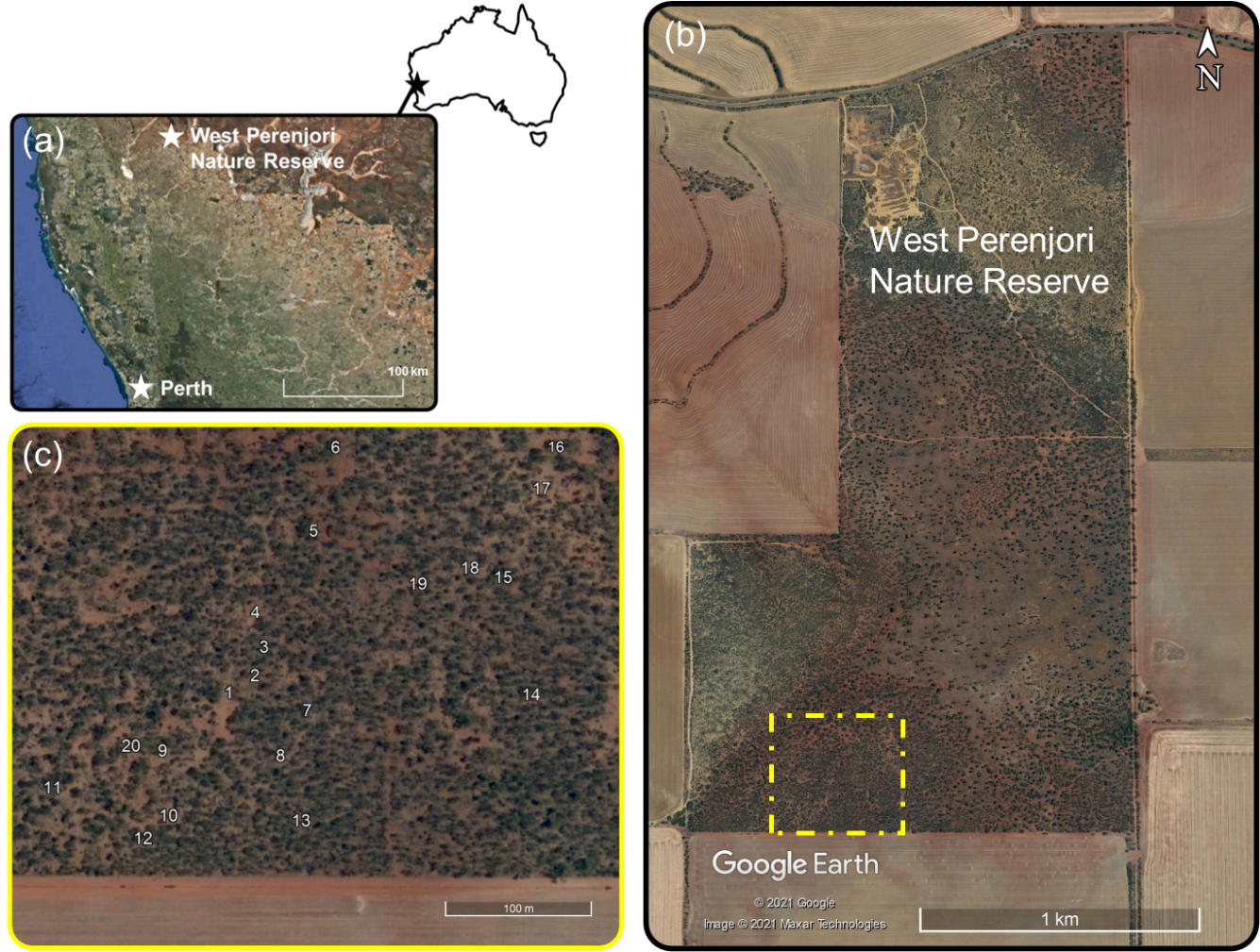 |
| --- |
| Figure S1: Hierarchical representation of the study design. The study was conducted (a) in West Perenjori Nature Reserve, north-east of Perth in southwest Western Australia. (b) West Perenjori Nature Reserve is a remnant patch in a matrix of neighbouring agricultural land use. The study was conducted within the area marked by the dashed yellow line. (c) The location of the seed bag burial sites. Note the sparse distribution of overstorey canopy cover. |

| 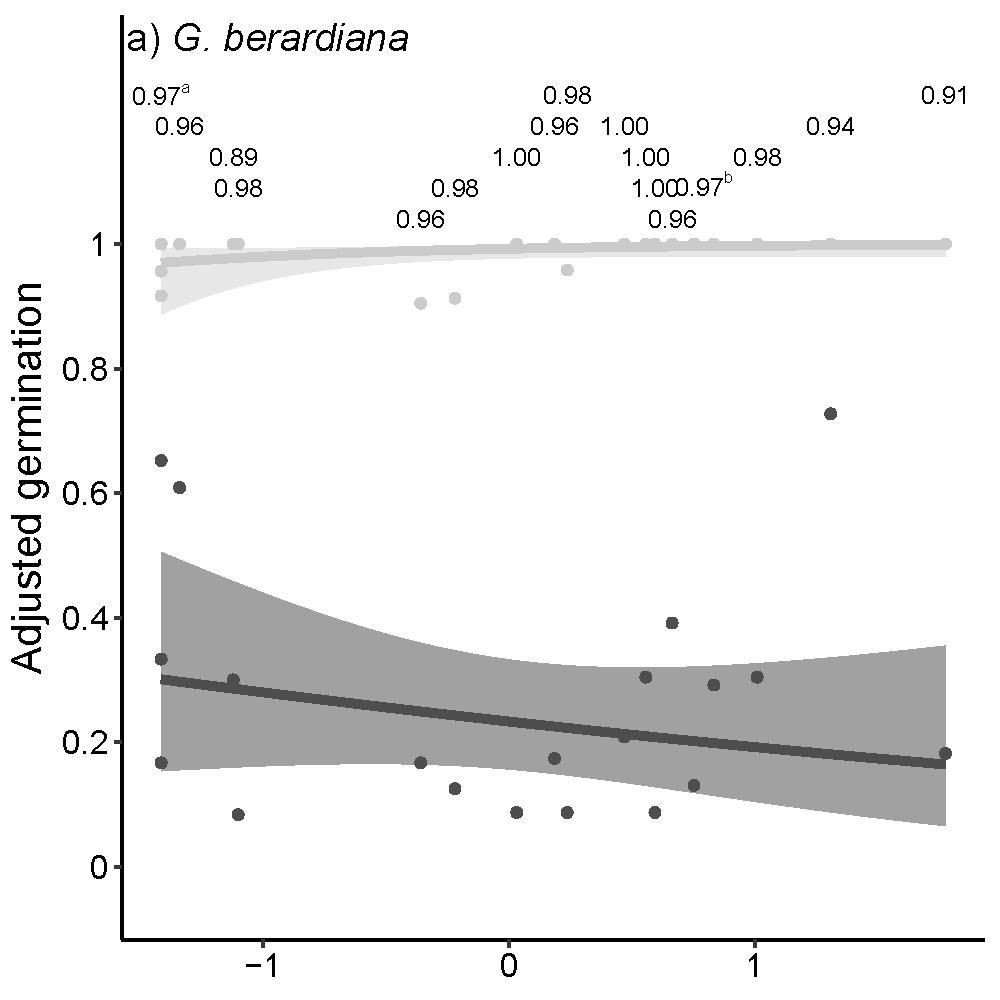 |
| --- |
| Figure S2: Relationship between species adjusted germination percentages and scaled sqrt(Seed bank canopy cover) in light (12h diurnal light) or dark conditions. Grey points represent adjusted germination percentages under light conditions while black points represent dark conditions. Solid lines represent the fitted relationship while the grey fields represent the 95% credible interval around the fitted relationship. |

| 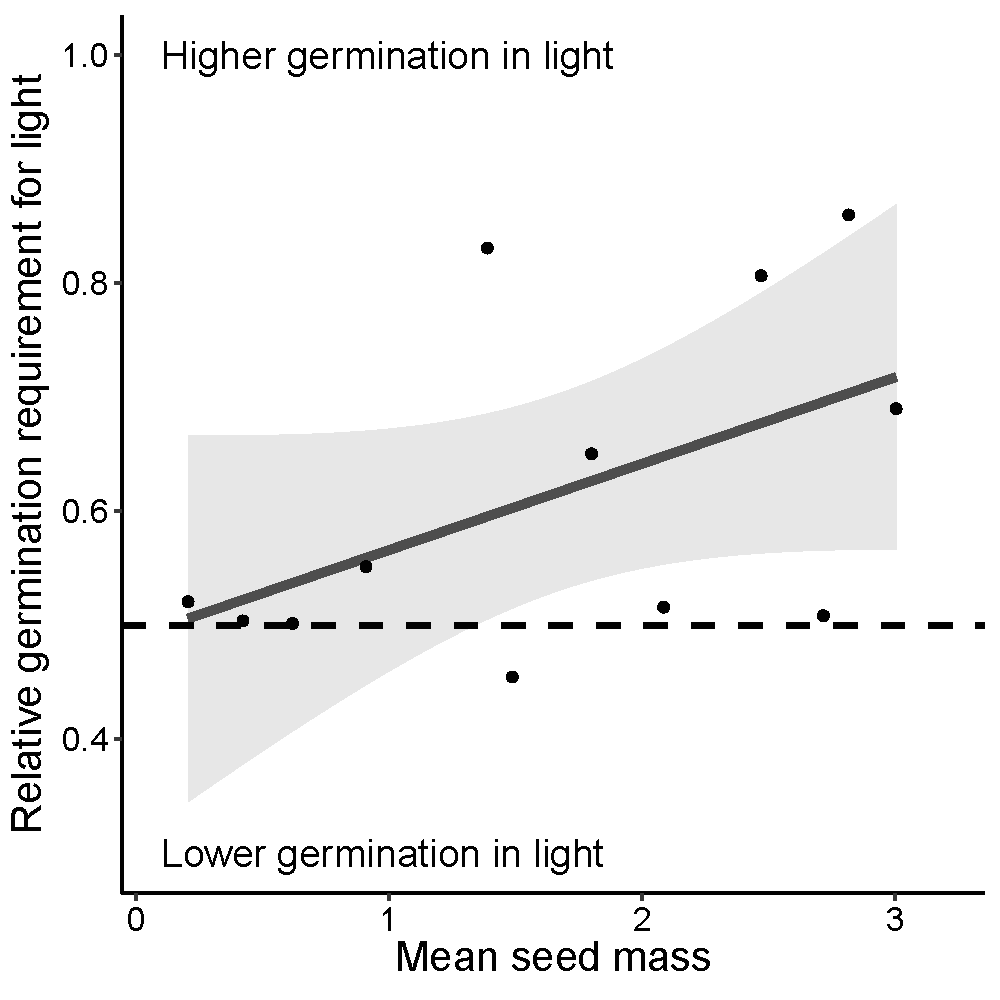 |
| --- |
| Figure S3: Relationship between species’ relative germination requirement for light (RLG) and species-level mean seed mass. Solid lines represent the fitted relationship while the grey fields represent the 95% credible interval around the fitted relationship. RLG values above the dashed line indicate higher germination under light than dark and *vice versa* below the dashed line. |
